# Synergistic effect of *Salvadora persica* and chitosan nanoparticles against oropharyngeal microorganisms

**DOI:** 10.1038/s41598-024-63636-1

**Published:** 2024-06-06

**Authors:** Hanan Balto, Mounir Salim Bekhit, Sayed H. Auda, Afaf Elansary, Ramesa Shafi Bhat, Najat Marraiki, Solaiman Al-Hadlaq

**Affiliations:** 1 Department of Restorative Dental Sciences, College of Dentistry, King Saud University, P.O. Box 62645, 11595 Riyadh, Saudi Arabia; 2 Department of Pharmaceutics, College of Pharmacy, King Saud University, 12372 Riyadh, Saudi Arabia; 3 Central Research Laboratory, Female Campus, King Saud University, Riyadh, Saudi Arabia; 4 Department of Biochemistry, College of Science, King Saud University, 11451 Riyadh, Saudi Arabia; 5 Department of Botany and Microbiology, College of Science, King Saud University, P.O. Box 2455, 11451 Riyadh, Saudi Arabia

**Keywords:** Drug discovery, Microbiology, Health care

## Abstract

Herbal medicine combined with nanoparticles has caught much interest in clinical dental practice, yet the incorporation of chitosan with *Salvadora persica* (*S. persica*) extract as an oral care product has not been explored. The aim of this study was to evaluate the combined effectiveness of *Salvadora persica*(*S. persica*) and Chitosan nanoparticles (ChNPs) against oropharyngeal microorganisms. Agar well diffusion, minimum inhibitory concentration, and minimal lethal concentration assays were used to assess the antimicrobial activity of different concentrations of ethanolic extracts of *S. persica* and ChNPs against selected fungal strains, Gram-positive, and Gram-negative bacteria. A mixture of 10% *S. persica* and 0.5% ChNPs was prepared (SChNPs) and its synergistic effect against the tested microbes was evaluated. Furthermore, the strain that was considered most sensitive was subjected to a 24-h treatment with SChNPs mixture; and examined using SEM, FT-IR and GC–MS analysis. *S. persica* extract and ChNPs exhibited concentration-dependent antimicrobial activities against all tested strains. *S. persica* extract and ChNPs at 10% were most effective against *S. pneumoni*, *K. pneumoni*, and *C. albicans*. SEM images confirmed the synergistic effect of the SChNPs mixture, revealing *S. pneumonia* cells with increased irregularity and higher cell lysis compared to the individual solutions. GC–MS and FT-IR analysis of SChNPs showed many active antimicrobial phytocompounds and some additional peaks, respectively. The synergy of the mixture of SChNPs in the form of mouth-rinsing solutions can be a promising approach for the control of oropharyngeal microbes that are implicated in viral secondary bacterial infections.

## Introduction

The mouth is the main gateway to the body and contains a highly diverse microbiota, most of which are commensal. Under normal conditions, the microbiota is able to migrate from the oral cavity to the lungs; hence, the lungs are enriched with oral taxa^1^. El-Solh et al.^2^ demonstrated that respiratory pathogens from the lung of institutionalized elder patients requiring mechanical ventilation are often genetically indistinguishable from strains isolated from the oral cavity. Co-infections caused by bacteria or fungi in several influenza virus pandemics have increased nowadays, and bacterial complications have increased the morbidity and mortality of viral infections, including Covid-19^3^.

*S. pneumoniae* was isolated in almost 50% of the cases of hospital-acquired pneumonia^4^. It is the most common bacteria found in viral secondary bacterial infections and is mostly associated with high mortality and morbidity during influenza epidemics and pandemics^5^. A recent investigation demonstrated that poor oral hygiene was linked to an increase in the number of obligate anaerobes located in the pneumonia-affected lungs of the studied patient population^6^. Professional oral hygiene measures decrease the number of oral bacteria^7^, reduce the number of days of fever, inhibit the development of pneumonia^8^, reduce the frequency of nosocomial pneumonia by nearly 40%^9^, and reduce the mortality rate caused by pneumonia^10^. Several other oral care practices have been attempted, including tooth brushing and mouthwashes. Gargling is considered to have promising effects through the removal of oral/pharyngeal proteases that help viral replication^11^.

*Salvadora persica* (*S. persica*), locally called miswak, is a traditional toothbrush that belongs to the *Salvadoraceae* family and has demonstrated antimicrobial properties against bacteria, viruses, and fungi^12–14^. The anti-inflammatory and antimicrobial effects of *S. persica* extracts on cariogenic and periodontal pathogens led to their incorporation into oral hygiene products such as mouthwashes and toothpastes^15^. Almaghrabi^16^ demonstrated strong activity of extracts obtained from fruits, twigs, and roots of *S. persica* against *S. pneumoniae* wild-type strains. Recent findings showed that *S. persica* extract decreased the amount of oral pathogen colonization in mechanically ventilated patients^17^.

In recent years, nanotechnology has emerged as an interdisciplinary field undergoing rapid development as a powerful tool for various biomedical applications, especially as antimicrobial agents^18^. Many oral care products, especially mouthwash and toothpastes, contain nanoparticles with anti-microbial, anti-inflammatory, and remineralizing properties^19^ have been investigated in dental applications. Among the various existing nanomaterials, chitosan nanoparticles (ChNPs) showed excellent antibacterial, antiviral, and antifungal properties^20^.

Combining *S. persica* extract with ChNPs is a novel approach with the intent of producing a synergistic antimicrobial effect against a wide range of microorganisms. Since there is evidence suggesting a close relationship between ventilator-associated pneumonia and the microbial profile present in the oral cavity^21^, this combination might be used as a preventive measure to control potentially harmful oropharyngeal microorganisms, as well as a therapeutic agent for established infections.

The incorporation of ChNPs with *S. persica* extract as an oral care product has not been extensively explored. Therefore, the aim of this study was to evaluate the antimicrobial efficacy of the ethanolic *S. persica* extract and ChNPs mixture against oropharyngeal microorganisms.

## Materials and methods

### *Salvadora persica* extract preparation

The plant collection and use were in accordance with all the relevant guidelines. *S. persica* root sticks were obtained from the Jazan region of Saudi Arabia. The sticks were cut into small pieces and milled by an electric grinder. Fifty grams of *S. persica* powder were extracted by soaking in 500 mL of 70% ethanol on a shaker at room temperature for 24 h. The extract solutions were then filtered using Whatman No. 4 filter paper and transferred to a porcelain dish to evaporate at 40 °C. The solutions were completely evaporated, and the powdered extract was stored in a refrigerator (4 °C) until required.

### Chitosan nanoparticles (ChNPs)

Chitosan nanoparticles were purchased from Nanoshel-UK Ltd. and prepared using the ionic gelation method. Different concentrations of chitosan (0.1 to 0.5 g) were added to 100 mL of 1% l-ascorbic acid and mixed well using a magnetic stirrer.

### Microbial strain and inoculum preparation

Gram-positive strains of *Streptococcus pneumoniae* (*S. pneumonia* ATCC 49136), *Streptococcus mutans* (*S. mutans* ATCC 25175), *Staphylococcus aureus* (*S. aureus* ATCC 25923); gram-negative strains of *Klebsiella pneumonia (K. pneumonia* ATCC 700603), *Escherichia coli* (*E. coli* ATCC 35218), *Pseudomonas aeruginosa (P. aeruginosa* ATCC 27853); and fungal strains of *Candida albicans* (*C. albicans* ATCC 14053) and *Candida krusei* (*C. krusei* ATCC 14243) were included in the study. All test strains were provided by the Central Research Laboratory-Female Campus and the lab of Biotechnology, College of Pharmacy-King Saud University.

The microbial strain was cultured on freshly prepared tryptic soy broth (TSB) (OXOID, Hampshire, England) for 18 h at 36 ± 1 °C to an exponential phase. *Streptococcus* species were additionally cultured on Brain Heart Infusion Broth (BHIB) (Difco, Laboratories, Detroit, MI) with 5% CO_2_ and incubated at 35 ± 1 °C. The optical density (OD _620_) of the microbial mass was detected by determining the turbidity of the bacterial growth using a spectrophotometer. The inoculum density was adjusted using normal saline to produce a final count of 1.5 × 10^6^ CFU/mL. The stock solution of *S. persica* extract was dissolved in dimethyl sulfoxide (DMSO) to achieve a final concentration of 512 mg/mL.

### Antimicrobial testing

The antimicrobial activity of different concentrations (25, 50, and 100 mg/mL) of *S. persica* extract and ChNPs was evaluated using standard agar well diffusion with Streptomycin (30 μg) and Amphotericin B (10 μg) discs (Oxoid, UK) serving as positive controls against bacterial and fungal strains, respectively, while ethanol and propylene glycol solution served as a negative controls for *S. persica* extract and ChNPs, respectively. All experiments were conducted in triplets.

Minimum inhibitory concentration (MIC) was performed by a standard twofold broth dilution method. Chlorohexidine (CHX) and fluconazole (FLZ) were used as positive controls. All experiments were conducted in triplicate for each extract. The MIC was the lowest concentration of antimicrobial agent that inhibited observable microbial growth.

Minimum lethal concentration (MLC) was preformed using the standard microdilution method. The MLC was detected as the lowest concentration of *S. persica* extract or ChNPs that did not allow visible growth (99.9% killing) on the agar plates after the incubation period. All procedures and microbiological manipulations were carried out in a Class II biological safety cabinet.

### Antimicrobial evaluation of 10% *S. persica* and 0.5% ChNPs mixture (SChNPs)

Based on the findings of antimicrobial tests, the most effective antimicrobial concentration of *S. persica* (100 mg/mL = 10%) and the maximum concentration of ChNPs (5 mg/mL = 0.5%) that provide better viscosity have been used to prepare the SChNPs mixture using three formulae and evaluated against all the test strains. Propylene glycol was added as a solvent for the hydroalcoholic extract of *S. persica*, and l-ascorbic acid was added to acidify the medium to make it suitable for the solubility of ChNPs. In addition, l-ascorbic acid acts as an antioxidant to protect the preparation contents against oxidation (Table 1).

**Table 1:** Formulae of the *S. persica*, ChNPs, and the SChNPs mixture.

Sample #	Propylene glycol w/w (%)	l-Ascorbic acid w/w (%)	*S. persica* w/w (%)	ChNPs w/w (%)
ChNPs	10	1	–	0.5
*S. persica*	10	1	10	–
SChNPs	10	1	10	0.5

### Scanning *electron* microscope (SEM) evaluation

The most sensitive bacteria (*S. pneumonia*) was exposed to *S. persica*, ChNPs, and SChNPs mixture for 24 h. *S. pneumonia* grown without an extract was taken as a control. After incubation, cells were centrifuged at 5000 r/min for 10 min. The pellets were washed with sterile PBS and fixed with 2.5% glutaraldehyde and 2% paraformaldehyde for 4 h at 4 °C with an intermittent vortex. The cell biomass was fixed to a glass coverslip, and morphological changes in the cell wall were viewed under SEM (JEOL SEM, Filed Emission 7610F).

### Fourier-transformed infrared (FTIR)

FTIR spectroscopy (FT-IR, Agilent Technologies, Santa Clara, California, United States) was used to identify the functional groups presented in the *S. persica* extract, ChNPs, and the SChNPs mixture. FTIR was measured in the range of 4000–500 cm^−1^.

### Gas chromatography-mass spectrometry (GC–MS)

The phytochemical analysis of *S. persica* extract and the SChNPs mixture was accomplished on GC–MS equipment (PerkinElmer/Clarus500, United States).

### Statistical analysis

Results were documented using Microsoft Excel 365, and computations were run three times. The mean and standard deviation are calculated based on the results. The MICs were computed using the SPSS software program, 2001 (version 15.0, Chigao, IL, USA). The antimicrobial efficacy of the different tested compounds was examined using one-way analysis of variance (ANOVA). Results were considered statistically significant when P < 0.05.

## Results

### Antimicrobial testing

**Figure 1:**
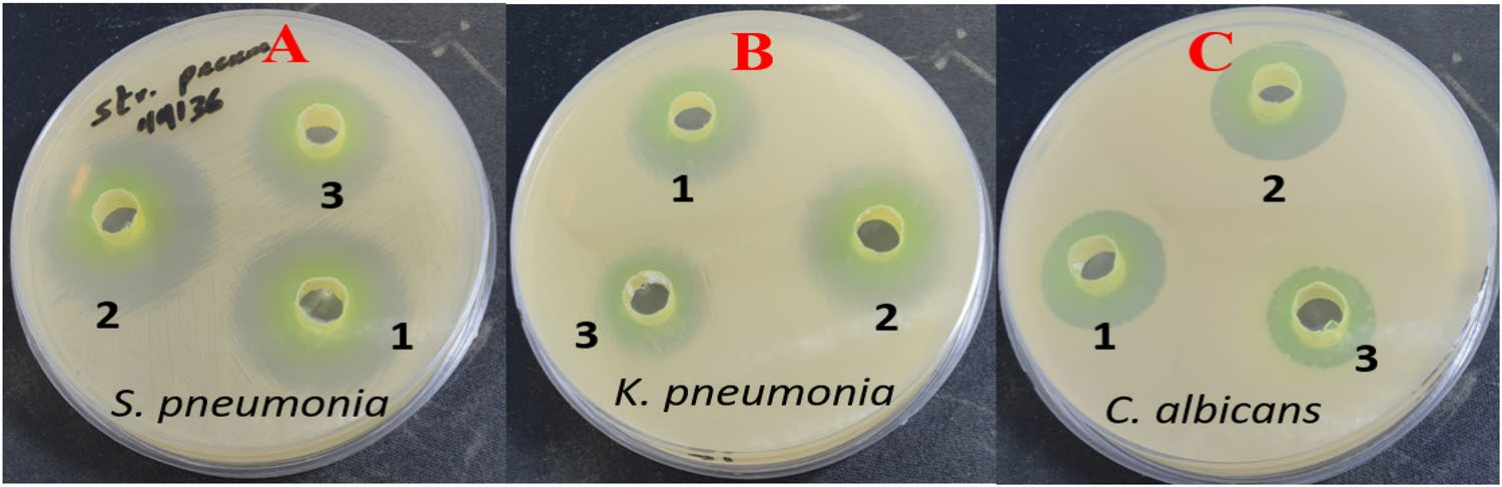
Zones of inhibition of *S. persica* alone (1) and in combination with ChNPs (2) against *S. pneumonia* (**a**); *K. pneumonia* (**b**) and *C. albicans* (**c**). The Strep. 30 μg and Amph. B was used as a positive control (3).

The findings showed the antimicrobial activity of *S. persica* extract and ChNPs (Fig. 1), this activity was concentration dependent. *S. persica* extract at 100 mg/mL was the most effective against all tested strains. The highest inhibition was observed against *S. pneumonia* and *S. mutans*. Overall, *S. persica* demonstrated more pronounced antimicrobial activity (inhibition zones ranged from 12.8 to 2.0 mm) against gram-positive strains (*S. pneumonia*, *S. mutans*, and *S. aureus*) than gram-negative strains (*K. pneumonia*, *P. aeruginosa*, and* E. coli*), where the inhibition zones ranged from 9.6 to 1.1 mm. In addition, *S. persica* extracts were effective against both tested candida species. The inhibition zones ranged from 9.3 to 1.6 mm in accordance with the *S. persica* concentration. With the 100 mg/mL concentration, *C. albicans* was most susceptible, followed by *C. krusei* (Table 2a).

**Table 2:** Antimicrobial activity profile of ethanolic extract of *S. persica* (a) and ChNPs (b) at different concentrations against gram-positive, gram-negative bacteria, and fungi (inhibition zone in mm).

	Inhibition zone (mm)							
	Bacterial strains						Fungi	
	Gram-positive			Gram-negative				
	*S. pneumonia*	*S. aureus*	*S. mutans*	*E. coli*	*K. pneumonia*	*P. aeruginosa*	*C. krusei*	*C. albicans*
(**a**)								
*Concentrations of S. persica extract*								
100 mg/mL (10%)	12.8 ± 0.01	10.4 ± 0.10	10.2 ± 0.20	9.0 ± 0.10	9.6 ± 0.10	9.1 ± 0.01	8.2 ± 0.03	9.3 ± 0.01
50 mg/mL (5%)	7.1 ± 0.03	6.5 ± 0.04	6.3 ± 0.02	6.6 ± 0.20	7.2 ± 0.02	7.0 ± 0.03	5.1 ± 0.00	5.3 ± 0.02
25 mg/mL (2.5%)	3.1 ± 0.02	3.7 ± 0.01	2.0 ± 0.00	3.9 ± 0.10	3.7 ± 0.01	1.1 ± 0.01	1.6 ± 0.00	1.9 ± 0.00
Strep. 30 μg	16.6 ± 0.00	15 ± 0.01	13.0 ± 0.00	16.0 ± 0.06	16.9 ± 0.07	15.0 ± 0.05	ND	ND
Amph. B (10 μg)	ND	ND	ND	ND	ND	ND	17.0 ± 0.02	17.6 ± 0.00
− ve control	NI	NI	NI	NI	NI	NI	NI	NI
(**b**)								
*Concentrations of ChNPs*								
100 mg/mL (10%)	11.2 ± 0.01	10.1 ± 0.01	10.8 ± 0.10	11.0 ± 0.02	11.3 ± 0.10	9.9 ± 0.01	11.5 ± 0.01	11.4 ± 0.03
50 mg/mL (5%)	10.4 ± 0.01	9.7 ± 0.00	10.1 ± 0.02	10.1 ± 0.01	10.3 ± 0.04	9.1 ± 0.01	10.3 ± 0.00	10.1 ± 0.02
25 mg/mL (2.5%)	6.0 ± 0.01	5.8 ± 0.03	6.2 ± 0.10	6.3 ± 0.02	6.1 ± 0.02	6.0 ± 0.04	7.0 ± 0.10	6.9 ± 0.02
Strep. 30 μg	16.6 ± 0.00	15.1 ± 0.01	13.0 ± 0.00	16.0 ± 0.06	16.9 ± 0.07	15.0 ± 0.05	ND	ND
Amph. B (10 μg)	ND	ND	ND	ND	ND	ND	17.0 ± 0.02	17.6 ± 0.00
− ve control	NI	NI	NI	NI	NI	NI	NI	NI

Microbial strain	100 mg/mL (10%) *S. persica*		100 mg/mL (10%) ChNPs		FLZ		CHX	
	MIC	MLC	MIC	MLC	MIC	MLC	MIC	MLC
(**c**)								
*S. pneumonia*	6.25 ± 0.0	12.5 ± 0.0	25 ± 0.0	50 ± 0.0	ND	ND	≤ 0.5	2
*S. mutans*	50 ± 0.0	100 ± 0.0	100 ± 14.4	> 100.00	ND	ND	≤ 0.5	2
*S. aureus*	25 ± 1.0	50 ± 1.4	50 ± 0.0	100.4.8	ND	ND	4	8
*E. coli*	25 ± 1.2	100 ± 0.0	100 ± 1.8	> 100.00	ND	ND	≤ 0.5	2
*K. pneumonia*	50 ± 0.2	50 ± 14.4	50 ± 0.0	100.04	ND	ND	8	16
*P. aeruginosa*	25 ± 0.0	50 ± 0.0	100 ± 0.0	> 100.00	ND	ND	8	16
*C. albicans*	6.25 ± 1.0	12.5 ± 0.0	25 ± 1.4	50 ± 2.0	≤ 0.5	25 ± 1.4	≤ 0.5	≤ 1.0
*C. krusei*	6.25 ± 0.0	12.5 ± 1.8	25 ± 0.0	50 ± 0.0	≤ 0.5	25 ± 1.4	≤ 0.5	≤ 1.0

Microbial strains	Tested compounds							
	*10% S. persica*		50% ChNPs		The mixture of 50% ChNPs and 10% *S. persica*		CHX	FLZ
	MIA 100 mg/mL	MIC 512 mg/mL	MIA 100 mg/mL	MIC 512 mg/mL	MIA 100 mg/mL	MIC 512 mg/mL	MIC mg/mL	
(**d**)								
*S. pneumonia*	12.8 ± 0.01	32	10.4 ± 0.01	128	24.1 ± 0.01	8	≤ 0.5	ND
*S. mutans*	10.2 ± 0.20	32	10.1 ± 0.02	128	23.8 ± 0.02	4	≤ 0.5	ND
*S. aureus*	10.4 ± 0.10	64	9.7 ± 0.00	128	21.9 ± 0.00	16	4	ND
*E. coli*	9.0 ± 0.10	128	10.1 ± 0.01	128	22.6 ± 0.01	16	≤ 0.5	ND
*K. pneumonia*	9.6 ± 0.10	256	10.3 ± 0.04	256	23.2 ± 0.00	32	8	ND
*P. aeruginosa*	9.1 ± 0.01	256	9.4 ± 0.01	256	21.6 ± 0.00	32	8	ND
*C. albicans*	9.3 ± 0.01	128	10.1 ± 0.02	128	24.5 ± 0.01	8	≤ 0.5	≤ 0.5
*C. krusei*	8.2 ± 0.03	128	10.3 ± 0.00	128	23.8 ± 0.00	8	≤ 0.5	≤ 0.5

2a—*Strep.* Streptomycin, *Amph. B* Amphotericin B, *NI* No inhibition, *ND* not detected.

2c—The mean values of MIC and MLC for the ethanol extracts of *S. persica *and ChNPs.Values are presented as mean ± standard deviation. *MIC* minimum inhibitory concentration, *MLC* minimum lethal concentration, *ND* not detected.

2d—Minimal inhibitory concentrations versus cup plate results of *S. persica*, ChNPs, and the SChNPs mixture against gram-positive, gram-negative bacteria, and fungi. *MIA* mean of inhibition area, *MIC* minimal inhibitory concentrations, *CHX* Chlorohexidine, *FLZ* Fluconazole, *ND* not detected.

ChNPs at 100 mg/mL were the most effective concentration against all tested microbial strains. The highest inhibition was observed against *S. pneumonia*, *E. coli*, *K. pneumonia*, and both strains of fungi. Overall, ChNPs demonstrated pronounced antimicrobial activity, with inhibition zones ranging from 11.2 to 5.8 mm against gram-positive strains and gram-negative strains. A similar inhibition zone (11.5–6.9 mm) was observed against both tested candida species (Table 2b).

The findings of MIC and MLC showed that *S. pneumonia* and both fungal strains were the most sensitive to the *S. persica* extract (6 and 12.5 mg/ml) and ChNPs (25 and 50 mg/ml), respectively (Table 2c).

The antimicrobial efficacy of the mixture (SChNPs) of 10% *S. persica* and 0.5% ChNPs against tested bacterial and fungal strains is presented in Table (2d). In all tested strains, the prepared mixture showed a significant zone of inhibition with a clear decrease in MIC. The range of the MIC was determined between 4 and 32 mg/mL. The maximum reduction was observed with *S. mutans* and *S. pneumonia* at 4 and 8 mg/mL, respectively. Additionally, it displayed good activity against *S. aureus* and *E. coli*, with MIC values of 16 mg/mL. Moreover, it exhibits good efficacy against *K. pneumonia* and *P. aeruginosa*, with MIC values of 32 mg/mL. The SChNPs  mixture also showed equivalently high activity against both fungal strains tested (*C. albicans* and *C. krusei*), with MIC values of 8 mg/mL.

### Scanning *electron* microscope evaluation

**Figure 2:**
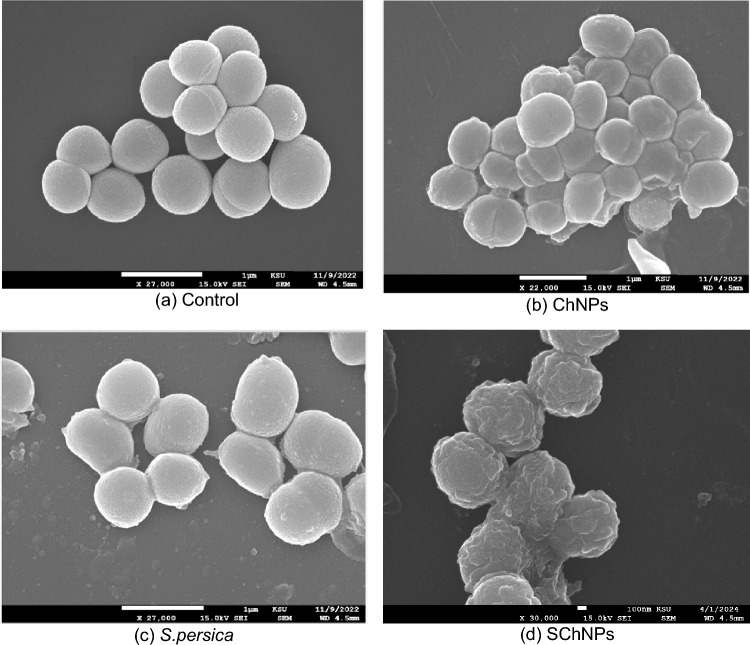
SEM micrograph showing the *S. pneumonia* cells: without any treatment as regular and smooth cocci (**a**); with aggregation and various levels of degeneration after 24 h treatment with ChNPs (**b**); *S. persica* (**c**); and the SChNPs mixture (**d**).

Scanning electron microscopy images of *S. pneumonia* treated with SChNPs showed some morphological changes as compared to the control (untreated *S. pneumonia* cells). Normal *S. pneumonia* cells were smooth, intact, and regular cocci (Fig. 2a), while ChNP-treated *S. pneumonia* showed slight irregularity in shape and aggregation (Fig. 2b). *S. persica*-treated cells showed minute irregularities in shape and clustering (Fig. 2c). Whereas SChNPs-treated cells showed remarkable morphological changes like blebs and destructed cells (Fig. 2d).

### Fourier-transform infrared spectroscopy (FT-IR)

**Figure 3:**
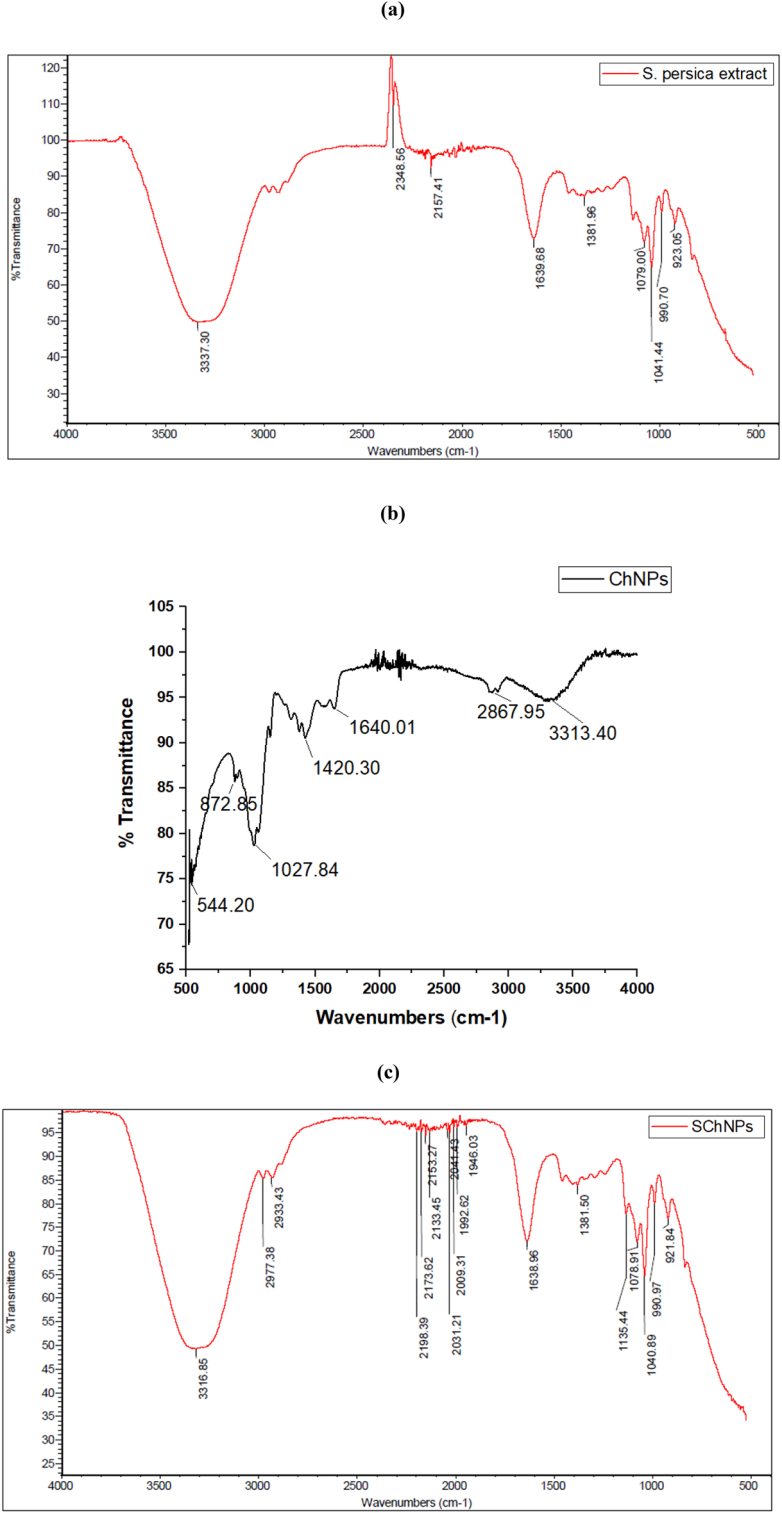
FT-IR spectra of *S. persica* extract (**a**); ChNPs (**b**); and the SChNPs mixture (**c**).

The FT-IR spectra peak of *S. persica*, ChNPs, and the SChNPs mixture are shown in Fig. 3a–c, and their probable functional groups are presented in Table 3. The spectrum shows many biologically active functional groups. FTIR spectrum results show the presence of aldehydes, alkenes, carboxyl nitrogen-containing group, aromatic primary amines, sulfoxides, amines, esters, ethers, and alcohols in both samples. However, some additional bands for ketones, carboxylic acids, and benzenes were observed in the SChNPs mixture. These functional groups mainly belong to many secondary metabolites such as phenols, alkaloids, flavonoids, saponins, terpenoids, and polyphenol.

**Table 3:** FT-IR spectra showing observed peaks and probable functional groups in *S. persica* extract, ChNPs, and the SChNPs mixture.

Wave number (cm^−1^)			Probable functional group
Mixture of SChNPs	ChNPs	*S. persica* extract	
3316.85		3337.30	N__H stretch (Amines
	3313.40		–NH and OH group stretch
2977.38			S, O__H stretch (Carboxylic acids), C__H stretch(Alkenes)
2933.43	2861.98		S, O__H stretch (Carboxylic acids), C__H stretch(Alkenes)
		2348	S, O__H stretch(Carboxylic acids), C__H stretch (Alkanes)
2198.39			C≡C stretch (Alkynes)
2173.62			(CO)–H (Aldehydes)
2153.27		2157	(CO)–H (Aldehydes)
2133.45			C≡C (Alkynes)
2041.43			C≡C alkyne
2031.21			C≡C alkyne
1992.62			C = O stretch (Ketone),
1946.03			C = C (Benzene)
1638.68		1639.68	N__H bend (Nitro compounds, Amides), C__C stretch (Amides), C¼O stretch (Carboxylic acid, Ketone), C¼C (Benzene, Alkenes)
	1640.01		–CH– and –CH_2_–(Amines)
1381.50		1381.96	C__N stretch (Amines), C__O stretch (Esters), C__O stretch (Ethers, Alcohols), O__H band (Carboxylic acids)
1078.91		1079.00	S = O stretch (Sulfoxides), C__N stretch (Amines),
1040.89		1041.44	C__O stretch (Esters, Ether, Alcohol), = C__H bend (Alkenes)
	1027.84		Polysaccharide structure of CH
990.70	872.85	990.70	C = C__H bend (Alkenes) (Pectin)
923.05		933.05	S¼O stretch (Sulfoxides), C__N stretch (Amines), C__O stretch (Esters, Ethers, Alcohols), ¼C__H bend (Benzene, Alkenes) (Cellulose)

### Gas chromatography-mass spectrometry

**Figure 4:**
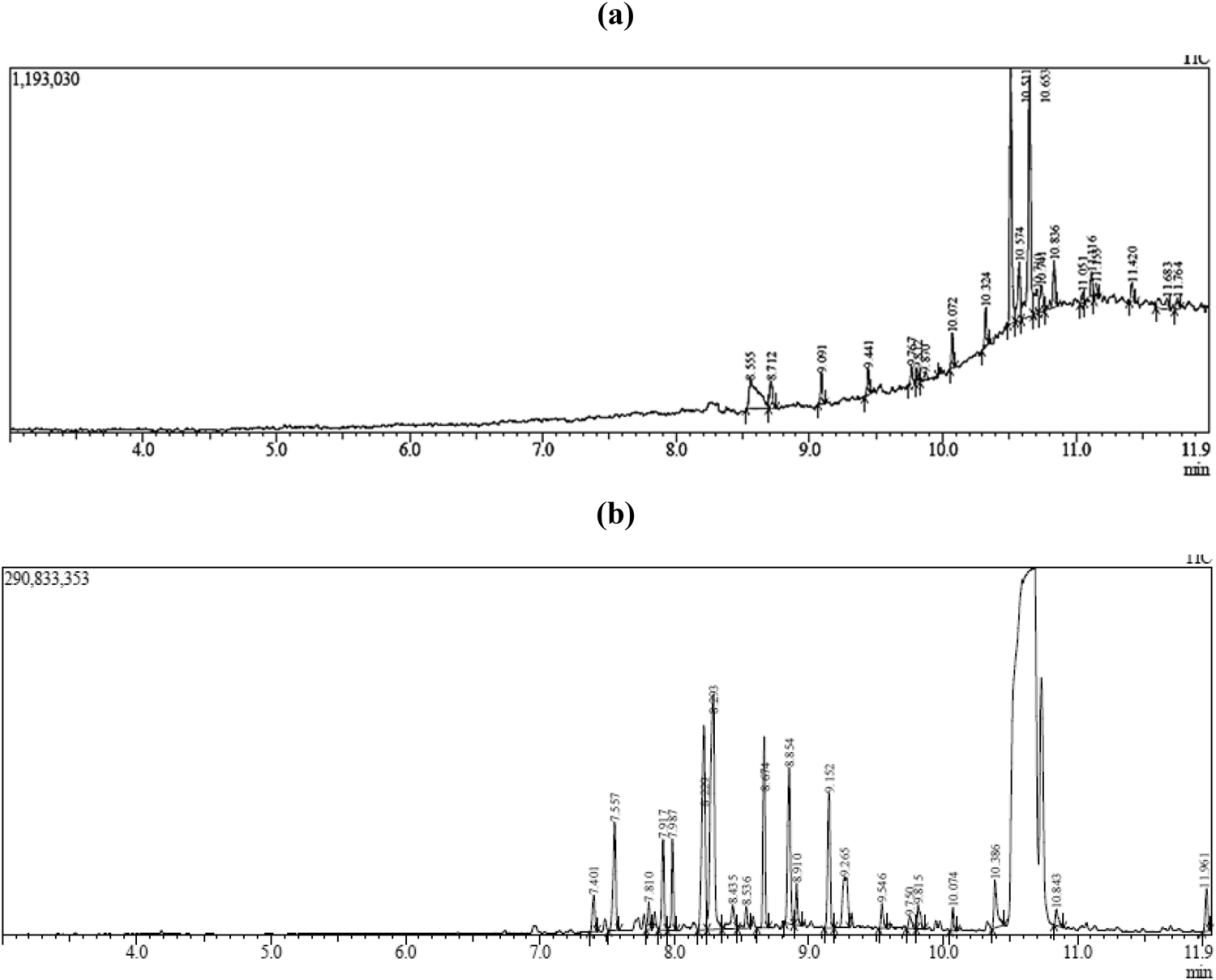
GC–MS analysis to detect anti-bacterial compound in *S. persica* extract (**a**) and the SChNPs mixture (**b**).

GC–MS chromatogram analysis of *S. persica*, and the SChNPs mixture is shown in Fig. 4 (a and b). Table 4a and b represent the list of major compounds identified by their retention time, molecular formula, and molecular weight.

**Table 4:** Bioactive compounds existing in *S. persica* extract (a) and the SChNPs mixture (b).

S. no	Name of the compound	Retention time (min)	Molecular formula	Molecular weight
(**a**)				
1	1-Hexadecanol	8.555	C_16_H_34_O	242
2	Octasiloxane,	8.721	C_16_H_50_O_7_Si_8_	578
3	Heptasiloxane	9.767	C_14_H_44_O_6_Si_7_	507
4	Sulfurous acid, 2-propyl undecyl ester	9.812	C_14_H_30_O_3_S	278
5	Cyclopropaneoctanoic acid	9.870	C_21_H_38_O_2_	322
6	Sulfurous acid, dodecyl 2-propyl ester	10.072	C_15_H_32_O_3_S_2_	292
7	Octacosane	10.32	C_28_H_58_	394
8	1,3-Benzenedicarboxylic acid, bis(2-ethylhexyl) ester	10.511	C_24_H_38_O_4_	390
9	Hexacosane	10.571	C_26_H_54_	366
10	Squalene	10.653	C_30_H_50_	410
11	2,6,10,14,18-Pentamethyl-2,6,10,14,18-eicos	10.710	C_25_H_42_	342
12	4,8,12-Tetradecatrien-1-ol, 5,9,13-trimethyl-	10.745	C_17_H_30_O	250
13	Heptacosane	10.86	C_27_H_56_	380
14	2,6-Lutidine 3,5-dichloro-4-dodecylthio-	11.05	C_19_H_31_C_l2_NS	375
15	Hexacosane	11.11	C_26_H_54_	366
16	Cholesta-3,5-diene	11.15	C_27_H_44_	368
17	Sulfurous acid, 2-propyl tridecyl ester	11.42	C_16_H_34_O_3_S	306
18	Strychane, 1-acetyl-20.alpha.-hydroxy-16-methylene-	11.68	C_21_H_26_N_2_O_2_	338
19	Di-n-decylsulfone	11.76	C_20_H_42_O_2_S	346
(**b**)				
1	N-p-toluenesulfonyl-l-threonine, benzyl ester	7.40	C_18_H_21_NO_5_S	363
2	Tetradecanoic acid	7.55	C_14_H_28_O_2_	228
3	Pentadecanoic acid	7.88	C_15_H_30_O_2_	242
4	n-Nonadecanol-1	7.98	C_19_H_40_O	284
5	6-Octadecenoic acid	8.22	C_18_H_34_O_2_	282
6	l-(+)-Ascorbic acid 2,6-dihexadecanoate	8.29	C_38_H_68_O_8_	652
7	Eicosanoic acid	8.43	C_20_H_40_O_2_	312
8	cis-10-Nonadecenoic acid	8.53	C_19_H_36_O_2_	296
9	1-Nonadecene	8.67	C_19_H_38_	266
10	9-Octadecenoic acid, (E)-	8.85	C_18_H_34_O_2_	282
11	Octadecanoic acid	8.91	C_18_H_36_O_2_	284
12	Phenol, 2,4'-isopropylidenedi	9.15	C_15_H_16_O_2_	228
13	Tris(2-hydroxyethyl)isocyanurate	9.26	C_9_H_15_N_3_O_6_	261
14	Octacosane	9.54	C_28_H_58_	394
15	Octicizer	9.75	C_20_H_27_O_4_P	362
16	Hexacosane	10.07	C_26_H_54_	366
17	1,3-Benzenedicarboxylic acid, bis(2-ethylhexyl)	10.38	C_24_H_38_O_4_	390
18	3-Dimethylaminopropyl methacrylamide	10.84	C_9_H_18_N_2_O	170
19	Cholesterol	11.9	C_27_H_4_6O	386

## Discussion

Nowadays, many plant-derived herbal medicines are being used in oral health care, mainly as antimicrobial, anti-inflammatory, and analgesic agents. Mouthwashes derived from medicinal plants have shown promising results in plaque and gum diseases due to the presence of active ingredients with antioxidant properties^22^. In this study, the ethanolic extracts of *S. persica* exhibited concentration-dependent antimicrobial activities against all tested strains, and the highest inhibition was observed against *S. pneumonia* and *S. mutans*. Several reports have also found extracts of *S. persica* to be effective antimicrobial agent^23^. Overall, the tested ethanolic extract demonstrated more pronounced antimicrobial activity against gram-positive strains than gram-negative strains. This difference is probably caused by the bacterial cell walls having different chemical compositions, especially in the outer membrane of lipopolysaccharides (LPS) structure^24^. LPS are large molecules consisting of a lipid and a polysaccharide that create a barrier to diffusion, making gram-negative bacteria more resistant to antimicrobials.

Nano-based dental materials are increasingly used clinically for oral hygiene due to their antimicrobial activity^19^. Chitosan is used in many commercial and dental products, such as toothpaste, enamel repair materials, dental restorative materials, coatings on implants, and adhesives^25^.

In this study, the antimicrobial activity of the SChNPs mixture, consisting of 10% *S. persica* ethanolic extract, 0.5% ChNPS, 1% l-Ascorbic acid, and 10% propylene glycol, was evaluated against the previously mentioned standard strains, and a synergistic effect was observed through the widening of the inhibition zone. The presence of different bioactive phytochemical compounds in the SChNPs mixture accounted for the broad-spectrum antimicrobial activities. As a result of the existence of bioactive compounds like alkaloids, tannins, flavonoids, phenols, terpenoids, and anthraquinones in the SChNPs mixture, broad-spectrum antimicrobial activities were detected. There are several possible mechanisms for how some secondary metabolites act against microorganisms: alkaloids may disrupt cellular walls and/or inhibit DNA replication^26^; tannins may deactivate microbial adhesions, enzymes, and transport proteins in cellular envelopes^27^; flavonoids may inhibit the synthesis of nucleic acids and energy metabolism of microbial cell membranes^28^; phenolic compounds and terpenoids may disrupt the microbial cell membranes^29^; anthraquinones act by increasing the superoxide anion levels and increasing the amount of oxygen molecules^30^. The antimicrobial properties of chitosan are believed to function through binding to negatively charged bacterial cell walls, altering membrane permeability, followed by attachment to DNA, inhibiting DNA replication, and finally causing cell death^31^.

The MLC/MIC ratio for the SChNPs  mixture was determined to define whether the pharmaceutical formula was bacteriostatic or lethal in the tested samples. It is generally considered that a MLC/MIC ratio over 4 equates to bacteriostatic effects, while lesser values indicate lethal effects^32^. Consequently, the SChNPs mixture showed lethal efficacy against *S. mutans*, *S. pneumonia*, *C. albicans*, and *C. krusei* ; but bacteriostatic effects on *S. aureus*, *E. coli*, *K. pneumonia*, and *P. aeruginosa*.

The effects of *S. persica* extract and ChNPs on the surface morphology of *S. pneumonia* were examined by SEM. No morphological changes were observed in untreated *S. pneumoniae* cells with clear cell wall structure; however, the results showed a special trend of changes in cells treated with *S. persica* extracts, ChNPs, and the SChNPs mixture. *S. pneumonia* cells treated with the mixture of 10% *S. persica* and 0.5% ChNPs exhibited a more irregular cell shape and a high degree of cell lysis as compared to the individual solution. These results explained the synergistic effect through induced morphologic alterations by the damaging bacterial membrane and leaked cytoplasmic material. SEM results are well explained by the occurrence of many active phytocompounds, which were detected by GC–MS analysis. Many studies have reported the antimicrobial activity of these active compounds, such as tetradecanoic acid, pentadecanoic acid, n-Nonadecanol-1^33^, 6-Octadecenoic acid , l-(+)-Ascorbic acid 2,6-dihexadecanoate^34^, Eicosanoic acid ,1-Nonadecene, 9-Octadecenoic acid^35^, which were not detected in *S. persica* extract alone. These results support the SEM findings that the SChNPs mixture induces maximum damage to the bacterial cells. The biological activity of any compound is influenced by its functional groups, which aid in evaluating the structure–function relationship of the bio-organic compound. FT–IR spectral analysis showed some additional peaks in the SChNPs mixture that were not detected in *S. persica* extract. FTIR analysis plays a crucial role in identifying and quantifying plant substances such as cell wall components, proteins, and lipids, aiding in understanding biochemical changes in samples. Changes in band height suggest modifications in chemical groups within lipids, carbohydrates, proteins, and cell wall regions^36,37^.

## Conclusion

Across all studied strains, the prepared mixture of 10% *S. persica* and 0.5% ChNPs (SChNPs) demonstrated a large zone of inhibition with a distinct decrease in MIC, indicating its synergistic effect. The use of SChNPs mixture as a mouthwash can act synergistically to control oropharyngeal microbes that are implicated in secondary micorbial infections associated with primary viral infections.

## Data Availability

All data generated or analyzed during this study are included in this published article.
